# Trends in Use, Outcomes, and Disparities in Endovascular Thrombectomy in US Patients With Stroke Aged 80 Years and Older Compared With Younger Patients

**DOI:** 10.1001/jamanetworkopen.2022.15869

**Published:** 2022-06-07

**Authors:** Amelia K. Adcock, Lee H. Schwamm, Eric E. Smith, Gregg C. Fonarow, Mathew J. Reeves, Haolin Xu, Roland A. Matsouaka, Ying Xian, Jeffrey L. Saver

**Affiliations:** 1 Department of Neurology, West Virginia University School of Medicine, Morgantown; 2 Department of Neurology, Massachusetts General Hospital, Harvard Medical School, Boston; 3 Department of Clinical Neurosciences and Hotchkiss Brain Institute, University of Calgary, Calgary, Alberta, Canada; 4 Division of Cardiology, University of California, Los Angeles; 5 Section Editor, Health Care Quality and Guidelines, *JAMA Cardiology*; 6 Department of Epidemiology, Michigan State University, East Lansing; 7 Duke Clinical Research Institute, Duke University, Durham, North Carolina; 8 Department of Neurology, University of California, Los Angeles

## Abstract

**Importance:**

Patients aged 80 years and older were often excluded or underrepresented in pivotal endovascular thrombectomy (EVT) trials. Accordingly, trends in frequency, outcomes, and disparities of EVT use merit close analysis.

**Objective:**

To delineate temporal trends in EVT use, outcomes, and disparities among patients with acute ischemic stroke aged 80 years and older vs those younger than 80 years.

**Design, Setting, and Participants:**

A US nationwide retrospective cohort study using prospectively collected data was conducted in patients admitted with a primary diagnosis of acute ischemic stroke between April 1, 2012, and June 30, 2019. Data were obtained from hospitals participating in the Get With the Guidelines–Stroke (GWTG-Stroke) program, which is a stroke quality improvement registry, with data collected prospectively, sponsored by the American Heart Association/American Stroke Association. Data analysis was conducted from November 2, 2020, to June 25, 2021.

**Exposures:**

Potentially eligible for EVT based on arrival within 6 hours and National Institutes of Health Stroke Scale score greater than or equal to 6.

**Main Outcomes and Measures:**

Efficacy outcomes included discharge to home, independent ambulation at discharge, and functional independence (modified Rankin Scale score 0-2) at discharge. Safety outcomes included in-hospital mortality, combined in-hospital mortality or discharge to hospice, and symptomatic intracranial hemorrhage.

**Results:**

Among 302 965 patients with ischemic stroke meeting study criteria as potentially eligible for EVT admitted to 614 GWTG-Stroke hospitals, 14.0% (42 422) received EVT (21 634 women [51.0%]), including 10.7% (12 768 of 119 453) of patients aged 80 years and older (median [IQR] age, 85 [82-89] years) and 16.2% (29 654 of 183 512) of patients younger than 80 years (median [IQR] age, 65 [56-73] years). Among patients aged 80 years and older, EVT rates increased substantially during the study period, from 3.3% in early 2012 to 20.8% in early 2019. By study end, the relative rate of EVT among eligible patients aged 80 years and older compared with those younger than 80 years increased from 0.49 (3.3% vs 6.7%) to 0.76 (20.8% vs 27.3%). Older patients had worse outcomes at discharge compared with younger patients, including discharge to home: 12.5% vs 31.1% (adjusted odds ratio [aOR], 0.43; 95% CI, 0.40-0.46), functional independence (modified Rankin Scale score 0-2): 10.9% vs 26.6% (aOR, 0.45; 95% CI, 0.41-0.49), and inpatient death or discharge to hospice, 34.5% vs 16.1% (aOR, 2.22; 95% CI, 2.09-2.36). Symptomatic intracranial hemorrhage rates did not differ significantly (6.9% vs 6.5%; aOR, 1.04; 95% CI, 0.94-1.14).

**Conclusions and Relevance:**

In this cohort study, use of EVT among individuals aged 80 years and older increased substantially from 2012 to 2019, although the rate remained lower than in younger patients. Although favorable functional outcomes at discharge were lower and combined mortality or discharge to hospice was higher in the older patients, the risk of symptomatic intracranial hemorrhage was not increased.

## Key Points

**Question:**

What are the frequency and outcomes of endovascular thrombectomy therapy in patients aged 80 years and older with acute ischemic stroke?

**Findings:**

In this cohort study of 302 965 patients with acute ischemic stroke, the frequency of endovascular thrombectomy in patients aged 80 years and older increased from 3.3% in 2012 to 20.8% in 2019. Among endovascular thrombectomy–treated patients, those aged 80 years and older less often achieved functional independence at discharge and more often died or were discharged to hospice care.

**Meaning:**

The findings of this study suggest that use of endovascular thrombectomy among individuals aged 80 years and older has increased substantially, with few achieving functional independence by discharge.

## Teaser

This cohort study examines changes in the use of endovascular thrombectomy for treatment of ischemic stroke in patients aged 80 years and older from 2012 to 2019.

## Introduction

The population of individuals aged 80 years and older in the US is expanding rapidly, projected to increase 2.5-fold over the next 3 decades, from 13.3 million in 2020 to 32.6 million in 2050.^1^ Age is the most important nonmodifiable risk factor for stroke and stroke incidence increases exponentially with advancing years. Acute ischemic strokes in older patients present with more severe neurologic deficits and larger infarct volumes and yield more disabling and fatal outcomes.^2,3,4^ Moreover, a greater proportion of ischemic strokes in individuals aged 80 years and older is due to large-vessel occlusion,^5,6^ in part because atrial fibrillation, a major cause of large-vessel occlusion in acute ischemic strokes, is 4 times as prevalent among individuals older than 80 years.^7^

Randomized clinical trials have reported the clinical benefit of endovascular thrombectomy (EVT) for patients younger than 80 years with acute ischemic strokes due to large-vessel occlusion presenting within 6 hours or with favorable penumbral profiles on imaging 6 to 24 hours from the onset of symptoms.^8,9^ However, patients aged 80 years and older were excluded from several of the landmark EVT trials and enrolled in only small numbers in the remainder (n = 198).^9^ As a result, these trials did not definitively characterize EVT outcomes among patients aged 80 years and older. The current American Heart Association acute stroke treatment guidelines reflect this uncertainty, particularly in patients aged 90 years and older, by recommending an individualized patient approach among that population.^10^ In addition, trends in the frequency of EVT use among patients aged 80 years and older, including changes in practice based on the results of pivotal trials, remain understudied. Accordingly, this study was undertaken to (1) delineate trends in EVT treatment frequency among potentially eligible patients aged 80 years and older compared with younger patients, (2) identify patient and hospital characteristics associated with EVT treatment among older patients, and (3) compare outcomes following EVT in patients aged 80 years and older vs younger patients.

## Methods

Get With the Guidelines–Stroke (GWTG-Stroke) is an ongoing, voluntary, continuous registry and performance initiative.^11^ Participating hospitals collect and enter deidentified patient-level data, including demographic characteristics; medical history; brain imaging findings; other diagnostic testing results; treatments, including intravenous thrombolysis and EVT; treatment times; outcomes; complications (including mortality); and discharge destination. Race and ethnicity information was collected from the GWTG dataset as demographic data without examination as a study variable. Trained hospital personnel ascertain consecutive patients admitted with stroke by prospective clinical identification, retrospective identification using *International Classification of Diseases* discharge codes per time frame of the revisions (2012-2019), or a combination. Deidentified patient data are entered into the GWTG-Stroke database using a web-based patient-management tool (IQVIA Technologies). Excellent reliability of the abstracted data has been reported.^12^ The US GWTG-Stroke program currently has more than 2000 participating hospitals and more than 5 million patient records. As of 2020, approximately 70% of all stroke admissions in the US were entered into the GWTG-Stroke database. Although large, urban, and teaching hospitals are overrepresented, patients in the GWTG-Stroke database have been reported to be clinically similar to the larger US Medicare stroke population.^13^ All participating sites receive either institutional review board approval to enroll cases in GWTG-Stroke without requiring individual patient consent under the Common Rule (45 CFR §46) or a waiver of authorization and exemption from subsequent review. This study was approved by the data analysis center at Duke University and was performed in accordance with Strengthening the Reporting of Observational Studies in Epidemiology (STROBE) reporting guideline for cohort studies.

### Patient Population

We analyzed data from GWTG-Stroke registry sites from April 1, 2012, to June 30, 2019, among sites reporting a minimum annual volume of 30 patients with stroke, program participation for 4 or more consecutive quarters, and medical history items missing for less than 25% of the patients. To determine the population potentially eligible for EVT, we included patients with final diagnosis of ischemic stroke; National Institutes of Health Stroke Scale (NIHSS) score greater than or equal to 6, indicating a more severe stroke and therefore more likely to involve larger occlusion; and time from last-known well to arrival under 6 hours. Patients were excluded who left against medical advice or were transferred to another acute care hospital, received EVT at another hospital, had inpatient-onset stroke, or were missing initial NIHSS scores or discharge destinations.

### Outcomes

In-hospital outcomes among patients who received EVT were compared between individuals aged 18 to 79 years and those aged 80 years and older. The 4 primary outcomes were successful reperfusion (defined as thrombolysis in cerebral infarction score 2b-3), discharge to home, independent ambulatory status at discharge, and functional independence (modified Rankin Scale Global Disability [mRS] score 0-2) at discharge. The range of the mRS scores is from 0 to 6, with 0 to 5 indicating increasing levels of disability and 6 indicating death. Safety outcomes were in-hospital mortality, combined in-hospital mortality and discharge to hospice, and symptomatic intracranial hemorrhage (sICH). Symptomatic ICH was defined as any clinical deterioration associated with a 4-point or more increase in the NIHSS score and imaging evidence of parenchymal hematoma, subarachnoid hemorrhage, or intraventricular hemorrhage 36 hours or less following EVT.

### Statistical Analysis

Data analysis was performed from November 2, 2020, to June 25, 2021. The proportion of potentially eligible patients undergoing EVT over time was tabulated and graphed. Baseline patient characteristics, comorbidities, laboratory data, medications before admission, and hospital characteristics are described using proportions for categorical variables and medians with 25th and 75th percentiles for continuous variables. Differences in characteristics between age groups were compared using χ^2^ tests for categorical variables and Wilcoxon rank sum tests for continuous variables. Factors associated with EVT use in the older patients were assessed using logistic regression with generalized estimating equations to account for within-hospital correlations.

Outcomes among patients receiving EVT were compared between older (≥80 years) and younger (<80 years) patients. Models were adjusted for both patient and hospital characteristics (eTable 1 in the Supplement). Multivariant analysis among the aged 80 years and older cohort for indicators of good (discharged to home or acute rehabilitation) or poor (discharged to skilled nursing facility or hospice, or in-hospital death) outcomes was performed. To explore the outcomes after approval of EVT for clinical indications in 2015, interactions with age 80 years and older and with stroke before and after 2015 were included.

Missing outcome variables and variables in univariant tables were not imputed. Sensitivity imputation with inverse probability weighting was used. Model covariate missingness and imputation are described in eTable 2 in the Supplement.

All significance tests were 2-sided, with *P* < .05 considered statistically significant. Because of the large sample size, small differences could be statistically but not clinically significant. Therefore, absolute standardized differences (multiplied by 100) were calculated to compare the characteristics in older vs younger patients; an absolute standardized difference greater than 10% was considered potentially meaningful and greater than or equal to 25% likely meaningful. All analyses were performed using SAS, version 9.4 (SAS Institute Inc).

## Results

**Figure 1.**
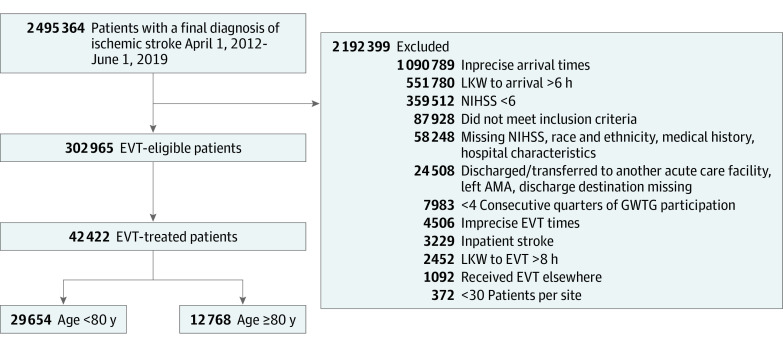
Patient Populations for Analysis AMA indicates against medical advice; EVT, endovascular thrombectomy; GWTG, Get With the Guidelines; LKW, last-known well; and NIHSS, National Institutes of Health Stroke Scale.

Between April 1, 2012, and June 30, 2019, 302 965 patients with ischemic stroke meeting study criteria as being potentially eligible for EVT were admitted to 614 GWTG-Stroke hospitals in the US (Figure 1). Among these, 42 422 patients (14.0%) were treated with EVT (21 634 women [51.0%], 20 788 men [49.0%]), including 10.7% (12 768 of 119 453) of patients aged 80 years and older (median [IQR] age, 85 [82-89] years; range, 80-105 years) and 16.2% (29 654 of 183 512) of patients younger than 80 years (median [IQR] age, 65 [56-73] years; range 18-79 years). Racial and ethnic groups comprised Asian (2286 [5.4%]); Black, non-Hispanic (6214 [14.6%]); Hispanic, any race (2887 [6.8%]); and White, non-Hispanic (29 429 [69.4%]) individuals. Among the EVT-treated patients, 12 768 (30.1%) were aged 80 years and older and 29 654 (69.9%) were younger than 80 years (Table 1). Considering each decade of life among the older EVT patients, 9999 (78.3%) were aged 80 to 89 years, 2716 (21.3%) were 90 to 99 years, and 53 (0.42%) were 100 years or older (eFigure 1 in the Supplement).

**Table 1.** Characteristics of Hospitals and Patients With Stroke Who Underwent Endovascular Thrombectomy

Variable	Age, y, No. (%)		Standardized difference
	<80 (n = 29 654)	80 and over (n = 12 768)	
Patient characteristics			
Age, median (IQR) [range], y	65 (56-73) [18-79]	85 (82-89) [80-105]	322.1^a^
Sex			
Women	13 203 (44.5)	8431 (66.0)	44.3^a^
Men	16 451 (55.5)	4337 (34.0)	
Race and ethnicity			
Asian	944 (3.2)	1342 (3.2)	31.3^a^
Black, non-Hispanic	5213 (17.6)	1001 (7.8)	
Hispanic (any race)	2121 (7.2)	766 (6.0)	
White, non-Hispanic	19 540 (65.9)	9889 (77.5)	
Other^b^	1836 (6.2)	714 (5.6)	
Patient location at onset^c^			
None	27 558 (93.7)	11 101 (87.6)	30^a^
Another acute care facility	1074 (3.7)	420 (3.3)	
Chronic health care facility	508 (1.7)	1034 (8.2)	
Outpatient health care setting	266 (0.9)	118 (0.9)	
Arrival by EMS	16 372 (55.3)	7742 (60.7)	11.1
Onset to arrival time, min	134 (58-217)	122 (54-210)	6.5
NIHSS initial score, median (IQR)	17 (12-22)	19 (14-23)	24.4
Ambulation status before admission^d^			
Independent	21 733 (96.5)	8460 (90.7)	14.7
With assistance (from person)	444 (2.0)	583 (6.3)	
Unable to ambulate	346 (1.5)	281 (3.0)	
Intravenous tPA use			
At EVT hospital	12 059 (40.7)	4940 (38.5)	4.6
At another hospital	7984 (39.4)	2747 (32.4)	14.7
Medical history			
Atrial fibrillation/flutter	7958 (26.8)	7108 (55.7)	61.3^a^
Coronary artery disease or myocardial infarction	6534 (22.0)	3600 (28.2)	14.2
Diabetes	7636 (25.8)	2920 (22.9)	6.7
Dyslipidemia	11 699 (39.5)	6180 (48.4)	18.1
Hypertension	19 857 (67.0)	10 253 (80.3)	30.6^a^
Chronic kidney disease	1599 (5.4)	1188 (9.3)	NA
Smoker	6604 (22.3)	379 (3.0)	60.7^a^
Prior stroke/TIA	5891 (19.9)	3187 (25.0)	12.2
Medications before admission			
Anticoagulant use	4889 (22.5)	3117 (37.8)	33.8^a^
Antiplatelet use	10 081 (38.1)	5747 (54.1)	32.5^a^
Physiologic measurements			
Systolic blood pressure, median (IQR), mm Hg	146 (129-165)	153 (136-173)	NA
BMI	29	26	55.3^a^
Hospital characteristics			
Academic	27 302 (92.1)	11 546 (90.4)	5.8
Stroke center status			7.3
Comprehensive stroke center	12 828 (43.3)	5065 (39.7)	NA
Primary stroke center	12 892 (43.5)	5864 (45.9)	
Neither	3934 (13.3)	1839 (14.4)	
Annual volume, median (IQR)			
Ischemic stroke	390 (280-528)	379 (274-527)	2.0
EVT	77 (48-117)	76 (48-116)	NA
Region^e^			11.5
Northeast	5910 (19.9)	2977 (23.3)	NA
Midwest	6236 (21.0)	2615 (20.2)	
South	12 080 (40.7)	4599 (36.0)	
West	5427 (18.3)	2577 (20.2)	

Abbreviations: BMI, body mass index (calculated as weight in kilograms divided by height in meters squared); EVT, endovascular thrombectomy; NA, not applicable; TIA, transient ischemic attack; tPA, tissue plasminogen activator.

^a^ Clinically meaningful difference.

^b^ American Indian/Alaska Native, Native Hawaiian/Pacific Islander, or unable to determine.

^c^ The denominator for number and percentage values is 29 406 patients for age less than 80 years and 12 673 patients for age 80 and older.

^d^ The denominator for number and percentage values is 22 523 patients for age less than 80 years and 9324 patients for age 80 and older.

^e^ The denominator for number and percentage values is 29 653 patients for age less than 80 years.

Patient- and hospital-level characteristics for patients aged 80 years and older and those younger than 80 years treated with EVT are reported in Table 1. Among EVT-treated patients aged 80 years and older, the median pretreatment NIHSS score was 19 (IQR, 14-23), 7622 (59.7%) received intravenous thrombolysis before EVT, and median time from last-known well to hospital arrival was 122 (IQR, 54-210) minutes. In addition, of patients with available data, 8460 of 12 768 (90.7%) were independent in ambulation before the stroke and 11 101 of 12 768 (87.6%) did not reside in a health care facility.

With regard to baseline characteristics, compared with younger individuals, more patients aged 80 years and older were women (843 [66.0%] vs 13 203 [44.5%]); were of White race (9889 [77.5%] vs 19 540 [65.9%]); were in a health care facility at symptom discovery (1454 [11.4%] vs 1582 [5.3%]); had a higher NIHSS score on presentation (median [IQR], 19 [14-23] vs 17 [12-22]); had a more frequent history of atrial fibrillation/flutter (7108 [55.7%] vs 7958 [26.8%]), hypertension (10 253 [80.3%] vs 19 857 [67.0%]), and prestroke treatment with antiplatelet agents or anticoagulant agents (8864 [69.4%] vs 14 970 [50.5%]); and were less often current smokers (379 [3.0%] vs 6604 [22.3%]) (Table 1). Differences in hospital characteristics between the age groups were not pronounced.

**Figure 2.**
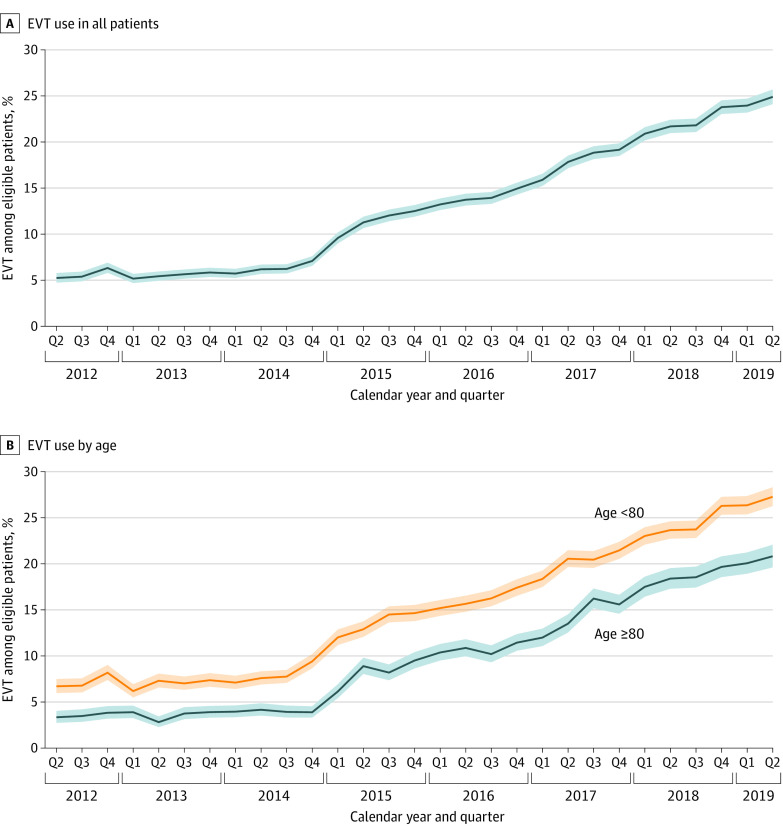
Trend in Rates of Endovascular Thrombectomy (EVT) Use Among Potentially Eligible Patients From 2012 to 2019 Use of EVT in all patients (A) and in patients aged 80 years and older and younger than 80 years (B). Shading represents 95% CIs.

Treatment rates with EVT in potentially eligible patients increased in both age groups throughout the study period, increasing in patients aged 80 years and older from 3.3% in quarter 3 of 2012 to 20.8% in quarter 2 of 2019 and in younger patients from 6.7% in quarter 3 of 2012 to 27.3% in quarter 2 of 2019 (Figure 2). Throughout the study period, the absolute rate of growth in both age groups was similar, although with patients aged 80 years and older starting from a lower baseline. As a result, the absolute difference in EVT rate between older and younger patients increased mildly, from 3.4% in quarter 2 of 2012 to 6.5% in quarter 2 of 2019; in contrast, the relative rate of EVT between older and younger patients increased markedly, from 0.49 to 0.76. Considering all ischemic stroke admissions of patients aged 80 years and older during the study period (720 151), not just those classified as potentially EVT eligible (119 453), the EVT rate increased from 1.1% in quarter 2 to quarter 3 in 2012 to 7.0% in quarter 1 to quarter 2 in 2019.

In both age groups, EVT treatment rate growth was faster in 2015-2019 compared with 2012-2014, coinciding with the landmark trials published in 2015 establishing EVT as the standard of care for large-vessel occlusion strokes. In formal analysis, an age and time period interaction was present, with the odds ratio (OR) for EVT in older compared with younger patients being 0.44 (95% CI, 0.40-0.48) before 2015 and 0.56 (95% CI, 0.54-0.58) after 2015; *P* < .001 for interaction). Rates of EVT in the potentially eligible older patients increased by 0.05% per year between 2012 and 2014 compared with 0.82% per year between 2015 and 2019.

**Figure 3.**
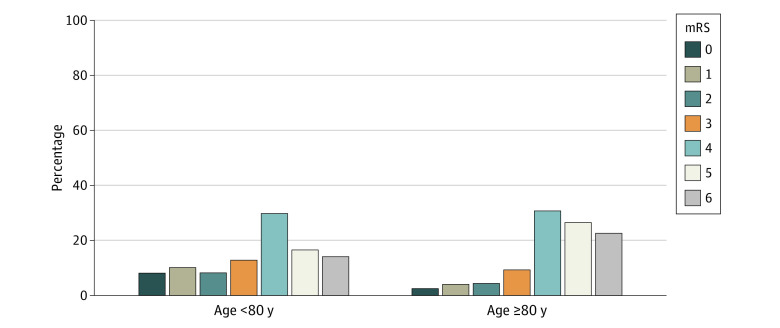
Distribution of Modified Rankin Scale (mRS) Global Disability Scores at Discharge Among Patients Aged 80 Years and Older and Younger Than 80 Years Scores from 0 to 5 indicate increasing levels of disability and 6 indicates death.

A technical outcome of successful reperfusion (thrombolysis in cerebral infarction score 2b-3) was achieved in 86.5% (8115 of 9385) among the older group compared with 88.4% (19 185 of 21 706) of the patients younger than 80 years (eTable 7 in the Supplement). Clinical outcomes in the older and younger age groups are reported in Table 2. Discharge to home was less frequent in older than younger patients (1591 [12.5%] vs 9222 [31.1%]; unadjusted OR, 0.32; 95% CI, 0.30-0.34; *P* < .001; adjusted OR [aOR], 0.43; 95% CI, 0.40-0.46). The other discharge destinations for patients aged 80 years and older were inpatient rehabilitation facility (3218 [25.2%]), skilled nursing facility (3256 [25.5%]), hospice or in-hospital death (4405 [34.5%]), and other (294 [2.3%]). Patients aged 80 years and older, compared with younger patients, were less frequently able to ambulate independently at discharge (2149 [17.9%] vs 10 216 [36.3%]; OR, 0.39; 95% CI, 0.37-0.41; *P* < .001). The older patients were also less frequently functionally independent (mRS score 0-2) at discharge (1032 [10.9%] vs 5854 [26.6%]; OR, 0.34; 95% CI, 0.31-0.37; *P* < .001). Across all levels of the mRS global disability scale, functional outcomes were less favorable in the older compared with younger patients (Figure 3). The adjusted odds ratios were higher than unadjusted odds ratios for all outcomes comparing patients aged greater than or equal to 80 years with patients aged younger than 80 years (eTable 5 in the Supplement).

**Table 2.** Clinical Efficacy and Safety Outcomes in Patients Who Underwent EVT^a^

Variable	Outcome rates		OR (95% CI)	
	Age <80 y	Age ≥80 y	Unadjusted	Adjusted
Efficacy				
Discharge home	9222 (31.1)	1591 (12.5)	0.32 (0.30-0.34)	0.43 (0.40-0.46)
Independent ambulation at discharge	10 216 (36.3)	2149 (17.9)	0.39 (0.37-0.41)	0.52 (0.48-0.55)
mRS 0-2 at discharge	5854 (26.6)	1032 (10.9)	0.34 (0.31-0.37)	0.45 (0.41-0.49)
Safety				
Symptomatic intracranial hemorrhage	1841 (6.3)	858 (6.9)	1.09 (1.00-1.19)	1.04 (0.94-1.14)
In-hospital mortality or hospice care	4780 (16.1)	4408 (34.5)	2.74 (2.61-2.89)	2.22 (2.09-2.36)

Abbreviations: EVT, endovascular thrombectomy; mRS, modified Rankin Scale; OR, odds ratio.

^a^ Models adjusted for sex, race and ethnicity, insurance status, symptom-onset location, ambulatory status before stroke, ambulatory status on admission, patient arrival method, medical history (atrial fibrillation/flutter, prosthesis heart valve, previous stroke/transient ischemic attack, coronary artery disease or prior myocardial infarction, carotid stenosis, diabetes, peripheral vascular disease, hypertension, smoker, dyslipidemia, heart failure), prior antiplatelets use, prior anticoagulant use, off-hour arrival, National Institutes of Health Stroke Scale score at admission, received intravenous tissue plasminogen activator (tPA) at this hospital or at an outside hospital, initial examination findings (weakness/paresis, altered level of consciousness, aphasia); rural location, region, stroke center status, teaching status, bed size, ischemic stroke volume, intravenous tPA volume, EVT volume, indicator variable for strokes before 2015; interactions of age 80 years and older, sex, and race and ethnicity with stroke before 2015.

Safety outcomes in the older and younger patients are reported in Table 2. Older patients had higher rates of combined inpatient death or discharge to hospice (4408 [34.5%] vs 4780 [16.1%]; OR, 2.74; 95% CI, 2.61-2.89; *P* < .001). In contrast, the rate of sICH did not differ significantly between older and younger patients (858 [6.9%] vs 1841 [6.3%]; OR, 1.09; 95% CI, 1.00-1.19; aOR, 1.04; 95% CI, 0.94-1.14).

Among patients aged 80 years and older, multivariant analysis estimated good outcome with the ability to ambulate independently or with minimal assistance at baseline, the use of intravenous tissue plasminogen activator, and the presence of aphasia at initial presentation. Factors estimating the probability of less favorable outcomes were older age, female sex, Black race, Hispanic ethnicity, stroke onset while in an acute or chronic care facility, previous stroke or transient ischemic attack, myocardial infarction or coronary artery disease, diabetes, peripheral vascular disease, higher NIHSS score, and altered level of consciousness at presentation (eTable 6 in the Supplement). There was no interaction observed between age, EVT success rate, and clinical outcome (eTable 2 in the Supplement). Among individuals aged 80 years and older, independent functional status decreased as age increased (eFigure 3 in the Supplement).

Among patients aged 80 years and older undergoing EVT, some outcomes improved from before to after the landmark trials were published in 2015 (eTable 4, eFigure 2 in the Supplement). Independent ambulation at discharge increased from 14.8% to 18.3% (OR, 0.73; 95% CI, 0.62-0.85; *P* < .001). In-hospital mortality decreased, but combined rates of in-hospital mortality and discharge to hospice did not change substantially. Rates of discharge to home, functional independence (mRS score 0-2) at discharge, and sICH also did not change markedly. Among the younger cohort, a somewhat similar temporal pattern of outcome evolution before and after 2015 was observed in all outcome measures, except improvements were noted in discharge to home and functional independence at discharge in the patients younger than 80 years (eTable 3, eFigure 2 in the Supplement).

## Discussion

In this study of nationwide stroke care within the US, the frequency of EVT performance in potentially eligible (<6 hours last-known well and NIHSS score ≥6), patients aged 80 years and older increased substantially between 2012 and 2019, from 1 in 25 patients to 1 in 5 patients. Considering all ischemic stroke admissions, during the same period the rate of EVT performance in the individuals aged 80 years and older increased from 1 in 100 patients at the start of the study period to 1 in 7 at its end. However, EVT procedures among patients aged 80 years and older continued to be performed less frequently than in younger patients, being conducted at three-fourths the rate in younger individuals. Although sICH was not increased in older patients, combined inpatient mortality or discharge to hospice was higher, occurring in 1 of 3 individuals aged 80 years or older. Overall, favorable outcomes at discharge were lower in the older cohort.

Among patients treated with EVT, the older and younger individuals differed most in several aspects. Characteristics associated with lengthened life span were more common in older patients: being female and not smoking. Age-related diseases and their treatments were more frequent: atrial fibrillation, hypertension, and antiplatelet and anticoagulant therapy prestroke. The older cohort also showed more frequent prestroke disability, prestroke residence in chronic care facilities, and higher NIHSS scores on hospital presentation.

The disparity in ischemic stroke EVT treatment rates between the older and younger patients observed in this study parallels that noted in rates previously reported for intravenous thrombolysis treatment in stroke and percutaneous angioplasty and stenting in myocardial infarction.^14,15,16^ However, this disparity diminished substantially over the 7-year observation period. Endovascular thrombectomy therapy rates among potentially eligible patients aged 80 years and older increased throughout the study period, with an accelerated increase after publication of pivotal clinical trials and national guideline endorsement in 2015. The pace of increase in EVT rates in the older population was 15-fold higher in 2015-2019 compared with 2012-2014. Although potentially eligible older patients received EVT half as often as younger patients at the start of the study period, by the end of the study the older cohort received EVT three-quarters as frequently. In addition, no plateau in EVT rates had been reached by the end of the study period, suggesting potential further increases in subsequent years. It is theoretically possible that the lower rates of treatment among the older group could be attributed to EVT barriers inherent to older patients (lower functional status, tortuous anatomy, and medical contraindications); however, the observation that the absolute difference in rates of use substantially changed over the study period is inconsistent with fixed barriers inherent to the patient population of interest.

Clinical outcomes were less favorable among patients aged 80 years and older who underwent EVT compared with younger patients. Only 1 in 8 older patients treated with EVT were discharged directly to home and only 1 in 9 were functionally independent at the time of discharge. However, because patients discharged to inpatient rehabilitation facilities generally are subsequently discharged to home 1 to 2 weeks later, the rate of patients aged 80 years and older treated with EVT eventually living at home was likely approximately 1 in every 2.7. Mortality was substantially higher after EVT in the older patients. Considering both inpatient deaths and discharges to hospice with expected early postdischarge fatality, approximately one-third of older patients treated with EVT had an early fatal outcome. The high mortality rate with EVT in patients aged 80 years and older is not as strong an impediment to intervention as it would be in younger patients. Often for older individuals, the worst possible outcome from acute stroke is surviving with severe disability; good functional outcomes or mortality may both be preferable to prolonged dependency.^17,18^ Observed outcomes improved mildly among older patients treated in the post-2015 era with expanded patient selection, likely reflecting increased interventionalist experience and expertise and advances in EVT device technology. Although rates of discharge mRS scores of 0 to 2 did not increase among the patients aged 80 years and older, rates of discharge mRS scores of 0 to 3 improved. The health-related quality-of-life value of an mRS 3 score outcome has been reported to be only mildly lower than for mRS 2 and mRS 0 to 3 scores have been defined as a good functional outcome among the oldest old EVT patients.^19,20,21^ Moreover, many patients discharged with an mRS score of 3 improve to an mRS of 1 to 2 by 90 days poststroke.^22^

The findings from the present study support and may extend the sparse available previous investigations. A recent meta-analysis identified only 3954 patients aged 80 years and older treated with EVT reported in 16 registry studies, most single-center.^23^ Four more recent studies have added 745 patients to this total.^24,25,26,27^ Accordingly, the current study’s population is more than double that of the previous reports combined and also reflects clinical practice in geographically diverse EVT treatment centers. Findings in the present study were similar to the meta-analyses and series reporting that older compared with younger patients who underwent EVT had a lower rate of functional independence, higher rate of mortality, and similar rate of sICH.^23,24,27^ The present study provides additional unique information regarding the full mRS outcome distribution, reperfusion status, discharge destination, and EVT rate changes over time.

The present study noted that age is an important prognostic marker in stroke, including among patients who undergo EVT. As a nonrandomized, observational cohort study, this investigation addresses age only as a prognostic marker (providing information about patient outcome regardless of therapy) and not as a predictive biomarker (providing information about whether a therapeutic intervention improves patient outcome).^28^ A pooled, individual participant data-level analysis of 5 randomized clinical trials suggested that EVT likely improves outcomes in the oldest age group.^9^ Among a total of 198 randomized patients, treatment with EVT compared with medical management alone yielded increased rates of functional independence (mRS score 0-2) at 3 months (29.8% vs 13.9%; relative ratio, 2.09; 95% CI, 1.03-4.25). Although the different timing of outcome assessment is a potential limitation in the present study (discharge) vs randomized trials (3 months) and precludes direct comparison of findings, the outcomes appear broadly comparable once account is taken of the inclusion of patients with preexisting disability before stroke and patients with multiple comorbidities in this clinical practice study who were excluded from randomized clinical trials.

### Limitations

This study has limitations. First, the data are dependent on the completeness and accuracy of medical record abstract information. The GWTG-Stroke program requires detailed training of site medical records abstractors and uses standardized case definitions and coding instructions, predefined logic/range checks on data fields, audit trails, and regular quality reports for all participating sites. Published audits have validated the reliability of data in GWTG-Stroke.^12^ Second, the population treated at GWTG-Stroke hospitals may not be fully representative of all patients treated in clinical practice. However, the GWTG-Stroke registry encompasses a substantial majority of all ischemic stroke admissions in the US and studies have reported good agreement between findings in the GWTG-Stroke registry and the general Medicare administrative database of all US patients older than 65 years,^12,13^ indicating good generalizability. Third, because of the absence of vessel occlusion site data in medically treated patients, the analysis used broad criteria when characterizing the potentially eligible EVT population, requiring only arrival under 6 hours last-known well and NIHSS score greater than or equal to 6. A more stringent NIHSS cutoff point greater than or equal to 10 would have increased specificity but reduced sensitivity in identifying potentially EVT-eligible patients.^29^ As a result, the number of patients designated as potentially EVT eligible in the present study is likely an overestimate. Future GWTG-Stroke studies will have a more accurate target population because the registry has recently added a field to capture the presence or absence of large-vessel occlusion in all patients, regardless of treatment administered. Fourth, patients aged 80 years and older in the present study were compared with younger patients whose ages span a large range, from 18 to 79 years, with potential within-group heterogeneity. Age-related differences in EVT use and response among subgroups of the 18-year to 79-year range will be the focus of a future study. Fifth, because the evidence supporting an expansion of EVT to the 6-hour to 24-hour window with imaging selection occurred in 2018,^30,31^ this study did not include patients in the extended thrombectomy period. Future investigation of trends in the use, outcomes, and disparities of use of EVT in older patients in this longer time window will be of interest.

## Conclusions

This study noted that use of EVT among patients aged 80 years and older with ischemic stroke has increased substantially, although treatment rates remain lower than in younger patients. Although the rates of favorable functional outcomes at discharge were lower and combined mortality and discharge to hospice were higher in the older patients, the risk of sICH is not increased. Patient outcomes in clinical practice were broadly consistent with those in randomized trials showing EVT benefit in the very old.

## Notes

Supplement. **eTable 1.** Variables Used in Adjusted Model **eTable 2.** Missingness Rates and Imputation Methods for Baseline Model Covariates **eTable 3.** Clinical Efficacy and Safety Outcomes in Younger (Age Under 80) Patients With Stroke Who Underwent EVT Before 2015 and After 2015 **eTable 4.** Clinical Efficacy and Safety Outcomes in Patients Aged 80 Years and Older With Stroke Who Underwent EVT Before 2015 and After 2015 **eTable 5.** Primary Model Results After Inverse Probability Weighting Adjustment for Missing Data **eTable 6.** Predictors of Favorable and Unfavorable Outcomes on Multivariant Analysis Among EVT Patients Aged 80 Years and Older **eTable 7.** Reperfusion Status and Clinical Outcome as a Function of Age **eFigure 1.** Histogram Chart of Number of Patients Aged 80 Years and Older Treated With and Without EVT in 5-Year Age Intervals **eFigure 2.** Distribution of mRS Global Disability Scores at Discharge Among EVT Patients Aged 80 Years and Older and Younger Than 80 Years Before 2015 and After 2015 **eFigure 3.** Distribution of mRS Global Disability Scores at Discharge in EVT Patients Aged 90 Years and Older
