# Supplementary material for: Trends in Use, Outcomes, and Disparities in Endovascular Thrombectomy in US Patients With Stroke Aged 80 Years and Older Compared With Younger Patients
Source: JAMA Netw Open. 2022 Jun 7;5(6):e2215869. doi: 10.1001/jamanetworkopen.2022.15869 (PMC9175073; doi:10.1001/jamanetworkopen.2022.15869)
Supplement: Supplement. — eTable 1. Variables Used in Adjusted Model eTable 2. Missingness Rates and Imputation Methods for Baseline Model Covariates eTable 3. Clinical Efficacy and Safety Outcomes in Younger (Age Under 80) Patients With Stroke Who Underwent EVT Before 2015 and After 2015 eTable 4. Clinical Efficacy and Safety Outcomes in Patients Aged 80 Years and Older With Stroke Who Underwent EVT Before 2015 and After 2015 eTable 5. Primary Model Results After Inverse Probability Weighting Adjustment for Missing Data eTable 6. Predictors of Favorable and Unfavorable Outcomes on Multivariant Analysis Among EVT Patients Aged 80 Years and Older eTable 7. Reperfusion Status and Clinical Outcome as a Function of Age eFigure 1. Histogram Chart of Number of Patients Aged 80 Years and Older Treated With and Without EVT in 5-Year Age Intervals eFigure 2. Distribution of mRS Global Disability Scores at Discharge Among EVT Patients Aged 80 Years and Older and Younger Than 80 Years Before 2015 and After 2015 eFigure 3. Distribution of mRS Global Disability Scores at Discharge in EVT Patients Aged 90 Years and Older [file jamanetwopen-e2215869-s001.pdf]

Supplementary Online Content

Adcock AK, Schwamm LH, Smith EE, et al. Trends in use, outcomes, and disparities in endovascular thrombectomy in US patients with stroke aged 80 years and older compared with younger patients. *JAMA Netw Open*. 2022;5(6):e2215869. doi:10.1001/jamanetworkopen.2022.15869

eTable 1. Variables Used in Adjusted Model

eTable 2. Missingness Rates and Imputation Methods for Baseline Model Covariates

eTable 3. Clinical Efficacy and Safety Outcomes in Younger (Age Under 80) Patients With Stroke Who Underwent EVT Before 2015 and After 2015

eTable 4. Clinical Efficacy and Safety Outcomes in Patients Aged 80 Years and Older With Stroke Who Underwent EVT Before 2015 and After 2015

eTable 5. Primary Model Results After Inverse Probability Weighting Adjustment for Missing Data

eTable 6. Predictors of Favorable and Unfavorable Outcomes on Multivariant Analysis Among EVT Patients Aged 80 Years and Older

eTable 7. Reperfusion Status and Clinical Outcome as a Function of Age

eFigure 1. Histogram Chart of Number of Patients Aged 80 Years and Older Treated With and Without EVT in 5-Year Age Intervals

eFigure 2. Distribution of mRS Global Disability Scores at Discharge Among EVT Patients Aged 80 Years and Older and Younger Than 80 Years Before 2015 and After 2015

eFigure 3. Distribution of mRS Global Disability Scores at Discharge in EVT Patients Aged 90 Years and Older

This supplementary material has been provided by the authors to give readers additional information about their work.

© 2022 Adcock AK et al. *JAMA Network Open.*

eTable 1. Variables used in adjusted model

Adjusted Variables
**Patient characteristics**
Age (≥ 80)
Sex
Race/ethnicity
White, non-Hispanic
Black, non-Hispanic
Hispanic (any race)
Asian
Other
Patient location at onset (home, acute care facility, chronic care facility, outpatient healthcare, other)
Arrival by emergency medical services
Off-hour arrival
Initial NIHSS Score, (0-42)
Exam findings (weakness/paresis, altered level of consciousness, aphasia)
Ambulation status prior to admission
Independent
With assistance (from another person)
Unable to ambulate
Received tPA at EVT or outside hospital
Medical history
Atrial fibrillation/Flutter
Prosthetic heart valve
Coronary artery disease or myocardial infarction
Heart failure
Carotid stenosis
Peripheral vascular disease
Diabetes mellitus
Dyslipidemia
Hypertension
Smoker
Prior Stroke/TIA
Medications prior to admission
Anticoagulant use
Antiplatelet use
**Temporal Trends**
Indicator variables for strokes prior to 2015
Interactions of age ≥ 80 stroke before 2015

© 2022 Adcock AK et al. *JAMA Network Open.*

Hospital characteristics
Teaching status (academic, non-academic)
Stroke Center status
Comprehensive Stroke Center (CRC)
Primary Stroke Center (PSC)
Neither CSC or PSC
Number of beds
Annual volume of ischemic stroke admissions
Annual volume of IVT
Annual volume of EVT
Rural location
Region
Northeast
Midwest
South
West

© 2022 Adcock AK et al. *JAMA Network Open.*

eTable 2. Missingness rates and imputation methods for baseline model covariates

Variable	Overall	EVT	No EVT	Imputation Details
Patient Demographics				
Age	0	0	0	N/A
Sex	0	0	0	N/A
Race/Ethnicity	0	0	0	N/A
Health Insurance Status	0	0	0	N/A
Year of Stroke Admission	0	0	0	N/A
Medical History	0	0	0	N/A
Ambulatory status prior to the current event	29.2	24.9	29.9	Missing/ND treated as a new category.
Arrival Information				
Ambulatory status on Admission	46.5	46.3	46.5	Missing/ND treated as a new category.
Patient location when stroke symptoms discovered	0.7	0.8	0.7	Missing treated as Not in a healthcare setting.
Arrival Mode: EMS	1.7	0.2	2.0	Missing treated as No.
Off-Hour Arrival*	0	0	0	N/A
Initial NIHSS Score (0-42)	0	0	0	N/A
Medications Prior to Admission				
Antiplatelets	12.1	12.6	12.0	Missing/ND treated as a new category.
Anticoagulants	40.0	29.3	41.8	Missing/ND treated as a new category.
Initial exam findings	33.0	30.7	33.3	Added an indicator variable for missing/ND.
Reperfusion				
IV t-PA at this hospital or at an outside hospital	0.0	0.0	0.0	Missing rate <.05, missing treated as No.
Hospital Characteristics				
Number of Beds	0	0	0	N/A
Rural Location	0	0	0	N/A
Stroke Center Status	0	0	0	N/A
Academic Hospital	0	0	0	N/A
Region	0	0	0	N/A
Annual Volume of IS Admissions	0	0	0	N/A
Annual Volume of IV t-PA	0	0	0	N/A
Annual Volume of EVT	0	0	0	N/A

*Regular Hour: 7A-6P, M-F, non-holiday)

© 2022 Adcock AK et al. *JAMA Network Open.*

eTable 3. Clinical efficacy and safety outcomes in younger (age under 80) patients with stroke who underwent EVT before 2015 and after 2015

	Before 2015	After 2015	P value
Efficacy			
Independent ambulation at discharge	1,140 (30.8)	9,076 (37.1)	<.0001
Discharged home	1,096 (27.3)	8,126 (31.7)	<.0001
mRS 0-2 at discharge	606 (21.5)	5,248 (27.3)	<.0001
Safety			
In-hospital mortality	471 (16.7)	2,653 (13.8)	0.0005
In-hospital mortality or hospice care	666 (16.6)	4,114 (16.0)	0.37
Symptomatic intracranial hemorrhage	252 (6.3)	1,589 (6.3)	0.99

© 2022 Adcock AK et al. *JAMA Network Open.*

eTable 4. Clinical efficacy and safety outcomes in patients aged 80 years and older with stroke who underwent EVT before 2015 and after 2015

	Before 2015	After 2015	P value
Efficacy			
Independent ambulation at discharge	186 (14.4)	1963 (18.3)	0.004
Discharged home	158 (11.2)	1433 (12.6)	0.13
mRS 0-2 at discharge	104 (10.3)	928 (11.0)	0.52
Safety			
In-hospital mortality	270 (26.9)	1848 (21.9)	0.0005
In-hospital mortality or hospice care	470 (33.3)	3938 (34.7)	0.31
Symptomatic intracranial hemorrhage	104 (7.4)	754 (6.8)	0.41

© 2022 Adcock AK et al. *JAMA Network Open.*

eTable 5. Primary model results after inverse probability weighting adjustment for missing data.

outcome	*Label*	*UORCI*	*P*	*AORCI*	*P*
O1: Discharged Home	Age >=80 vs <80	0.25 (0.13, 0.46)	<.0001	0.30 (0.20, 0.46)	<.0001
O2: Independent ambulation at discharge (among discharged alive patients)	Age >=80 vs <80	0.42 (0.30, 0.60)	<.0001	0.53 (0.38, 0.74)	0.0002
O5: mRS at discharge (0-2)	Age >=80 vs <80	0.30 (0.20, 0.45)	<.0001	0.45 (0.35, 0.58)	<.0001
O4: Symptomatic intracranial hemorrhage	Age >=80 vs <80	3.28 (1.56, 6.92)	0.0018	1.93 (1.24, 3.02)	0.0038
O3: In-hospital mortality/Discharged to Hospice	Age >=80 vs <80	5.38 (2.35, 12.29)	<.0001	3.96 (2.27, 6.90)	<.0001

© 2022 Adcock AK et al. *JAMA Network Open.*

eTable 6. Predictors of favorable and unfavorable outcomes on multivariant analysis among EVT patients aged 80 years and older

Variable	OR
Age, every 5-year increase	0.47 (0.43, 0.51)
Female	0.90 (0.83, 0.98)
Black	1.27 (1.09, 1.47)
Hispanic (any race)	1.23 (1.02, 1.49)
White	Reference
Another acute care facility	0.76 (0.61, 0.94)
Chronic health care facility	0.26 (0.21, 0.32)
Not in a healthcare setting	Reference
Able to ambulate independently	1.69 (1.19, 2.40)
Able to ambulate independently	1.54 (1.19, 1.99)
With assistance (from person)	2.02 (1.71, 2.40)
Previous Stroke/TIA	0.84 (0.76, 0.92)
Previous MI/CAD	0.88 (0.80, 0.96)
Diabetes Mellitus	0.72 (0.66, 0.79)
NIHSS, every 5-point increase	0.72 (0.69, 0.74)
Received IV tPA	1.13 (1.04, 1.24)
Altered level of consciousness	0.74 (0.67, 0.82)
Aphasia	1.29 (1.13, 1.47)
Western Region	0.74 (0.61, 0.91)
Northeast	Reference

© 2022 Adcock AK et al. *JAMA Network Open.*

eTable 7. Reperfusion status and clinical outcome as a function of age.

Age	Successful Reperfusion (mTICI 2b-3)	N	mRS at discharge (0-2) n/N (%)			In-hospital mortality/ Discharged to Hospice n/N (%)		
EVT patients age 18-79	1 = Yes	19185	4472	14735	30.35	2624	19185	13.68
EVT patients age 18-79	0 = No	2521	148	1916	7.72	772	2521	30.62
EVT patients age 80+	1 = Yes	8115	786	6176	12.73	2410	8115	29.70
EVT patients age 80+	0 = No	1270	21	942	2.23	773	1270	60.87

© 2022 Adcock AK et al. *JAMA Network Open.*

eFigure 1. Histogram chart of number of patients aged 80 years and older treated with and without EVT in 5-year age intervals

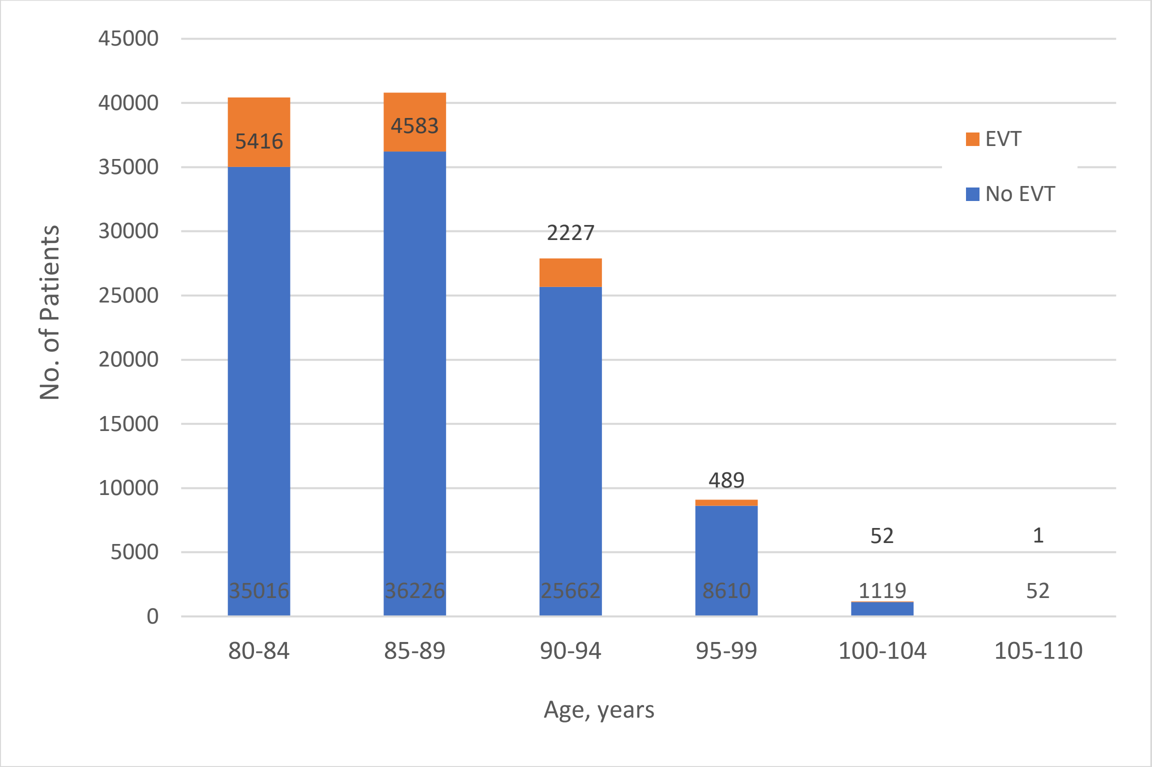

© 2022 Adcock AK et al. *JAMA Network Open.*

eFigure 2. Distribution of mRS global disability scores at discharge among EVT patients aged 80 years and older and younger than 80 years before 2015 and after 2015.

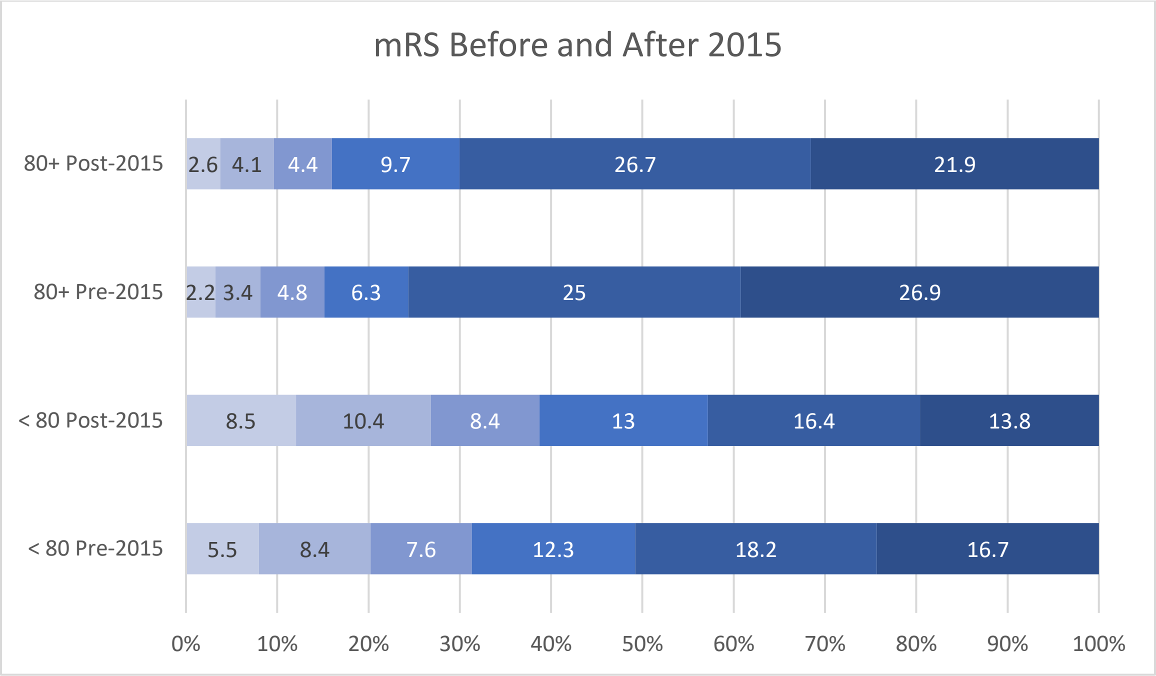

© 2022 Adcock AK et al. *JAMA Network Open.*

eFigure 3.Distribution of mRS global disability scores at discharge in EVT patients aged 90 years and older.

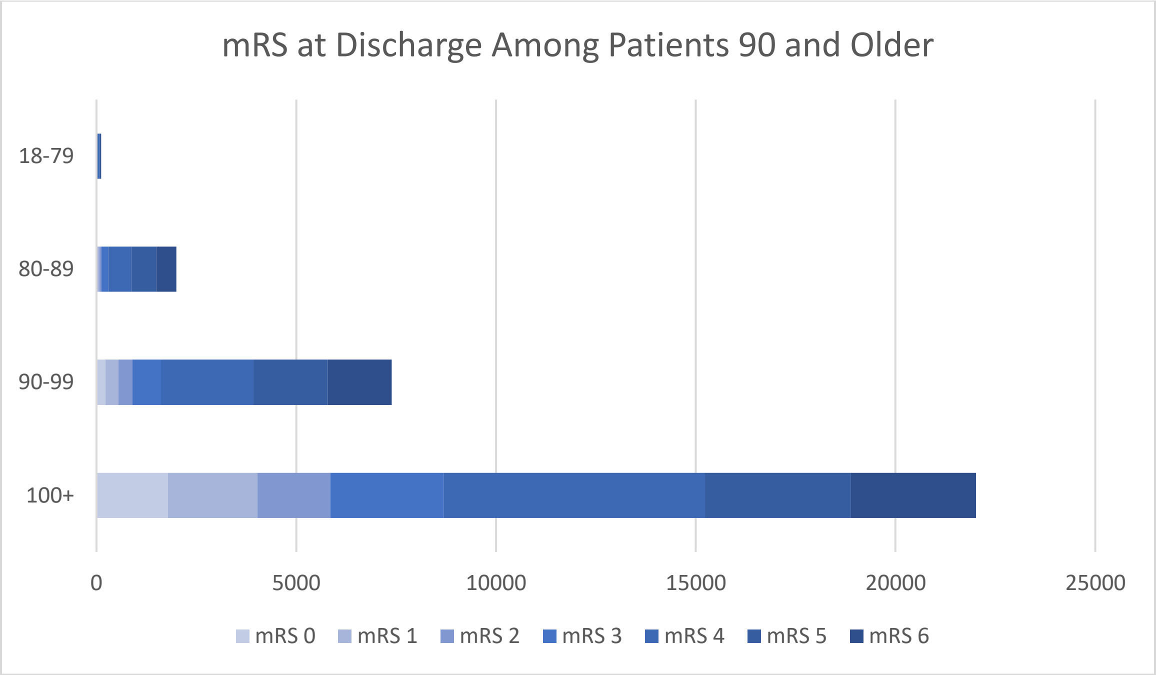

© 2022 Adcock AK et al. *JAMA Network Open.*
